# Past drivers of and priorities for child undernutrition in South Asia: a mixed methods systematic review protocol

**DOI:** 10.1186/s13643-019-1112-7

**Published:** 2019-07-31

**Authors:** Nidhi Wali, Kingsley Agho, Andre M. N. Renzaho

**Affiliations:** 1 School of Social Sciences and Psychology, Western Sydney University, Locked Bag 1797, Penrith, NSW 2751 Australia; 2 School of Science and Health, Western Sydney University Campbelltown Campus, Locked Bag 1797, Penrith, NSW 2751 Australia

**Keywords:** Child undernutrition, Determinants, Micronutrient deficiencies, South Asia, Stunting, Underweight, Wasting

## Abstract

**Background:**

South Asia has one of the largest proportions of undernourished children in the world, especially stunting, wasting, and underweight as well as micronutrient deficiencies such as the deficiency of iron, vitamin A, and zinc. Undernutrition continues to pose a major threat to this region’s economic and social growth. This systematic review aims to assess the drivers and identify priorities for child undernutrition in South Asia. It aims to appraise, synthesise, and summarise literature to create an evidence base that looks at multiple faces of macro and micro child undernutrition in South Asia.

**Methods:**

A systematic review of published and grey literature on child undernutrition, including macro and micronutrient deficiencies, in South Asia covering the period January 2000 to September 2019 will be undertaken. Studies with all relevant study designs and those published in English will be considered for inclusion. Five academic databases will be searched: CINAHL, EMBASE, PubMed, PsycINFO, and Scopus, in addition to various grey literature sources. The analysis will incorporate a narrative synthesis, meta-ethnography or a meta-analysis as appropriate, depending on the nature of the retrieved data. Quality of the included studies will be assessed by validated tools. The UNICEF conceptual framework on child undernutrition will be used to frame findings.

**Discussion:**

This protocol is guided by the Preferred Reporting Items for Systematic Reviews and Meta-Analyses Protocols (PRISMA-P) guidelines. The protocol gives an insight into the scope and parameters for the systematic review to be carried out.

**Systematic review registration:**

The protocol was registered by the PROSPERO international prospective register of systematic reviews, reference CRD42018112696.

**Electronic supplementary material:**

The online version of this article (10.1186/s13643-019-1112-7) contains supplementary material, which is available to authorized users.

## Background

Child undernutrition continues to be a major public health concern [1] and is considered an underlying cause of around 60% of deaths amongst children under 5 years of age in low- and middle-income countries (LMICs) [2]. Undernutrition encompasses macronutrient (fats, protein, and carbohydrate) and micronutrient (minerals and vitamins) deficiencies. Malnutrition encompasses both undernutrition (stunting, wasting, underweight and deficiencies of essential vitamins and minerals) and overnutrition (obesity or over-consumption of specific nutrients) [3]. Undernutrition has dire consequences for the health of children and the long-term economic productivity of nations [4–6]. It is associated with increased morbidity and mortality due to infections, increased risk of maternal, perinatal and neonatal mortality and increased risk of chronic diseases in adults. It is also associated with poor child development leading to poor school performance with long-term economic and social implications [6, 7].

Global estimates of undernutrition amongst children under 5 years of age suggest that in 2017, 151 million children were stunted, 50.5 million children were wasted [8] and 90 million children were underweight [9]. In addition, deficiencies of essential vitamins and minerals such as iron, vitamin A, and zinc affect an estimated two billion people or almost one-third of the world’s population [5]. These micronutrient deficiencies are often referred to as ‘hidden hunger’ or ‘hidden malnutrition’ as they may invisibly affect the health and development of a population [10]. Many countries have a high prevalence of more than one form of undernutrition. This multiple burdens of undernutrition are more prevalent in LMICs and concentrated amongst the poor [8].

### Child undernutrition in South Asia

South Asia and Sub-Saharan Africa have nearly 90% of the world’s underweight children where half live in South Asia [9]. South Asia also bears the highest prevalence globally with 35% of stunted children and wasting prevalence above the 15% threshold along with micronutrient deficiencies [6, 11], all which establish child undernutrition as a ‘critical public health problem’ in the region. Collectively India, Bangladesh, and Pakistan have the highest global levels of disability-adjusted life years (DALYs) attributable to child undernutrition [5]. The three micronutrient deficiencies of iron deficiency anaemia, vitamin A, and zinc, each has a global significance in South Asia [12, 13]. Prevalence of anaemia continues to be a severe public health problem (≥ 40%) in all South Asian countries amongst children of 6–59 months, with the exception of Sri Lanka [5]. Vitamin A deficiency (VAD) affects an estimated 44–50% of preschool children in South Asian regions. In Bangladesh and India, mortality due to VAD and undernutrition constituted one-third of the global mortality rate [14]. Inadequate zinc intake continues to be highly prevalent in South Asia and the zinc deficiency burden is reported to be very high in the region compared with other low-income regions [15]. For instance, zinc deficiency defined as serum zinc concentration < 60 μg/dl was reported to be prevalent amongst 15% of children under 5 years in Afghanistan while in Bangladesh and Pakistan, the prevalence is much higher at 45% and 39%, respectively [5].

South Asia presents a paradox, also commonly known as the ‘South Asia enigma,’ a term used to describe the persistence of high levels of child undernutrition despite economic growth in the region [16]. The region has become the fastest-growing developing region in the world with significant economic progress, marked poverty reduction, improved health indicators, improved literacy rates, and improved agriculture outputs [17–19]. This same region also hosts the largest burden on child undernutrition and micronutrient deficiencies along with rapidly increasing rates of child obesity that can have increased risk in adulthood of cardiovascular diseases, cancers, type 2 diabetes, and dementia [20, 21].

### UNICEF conceptual framework on child undernutrition

**Fig. 1:**
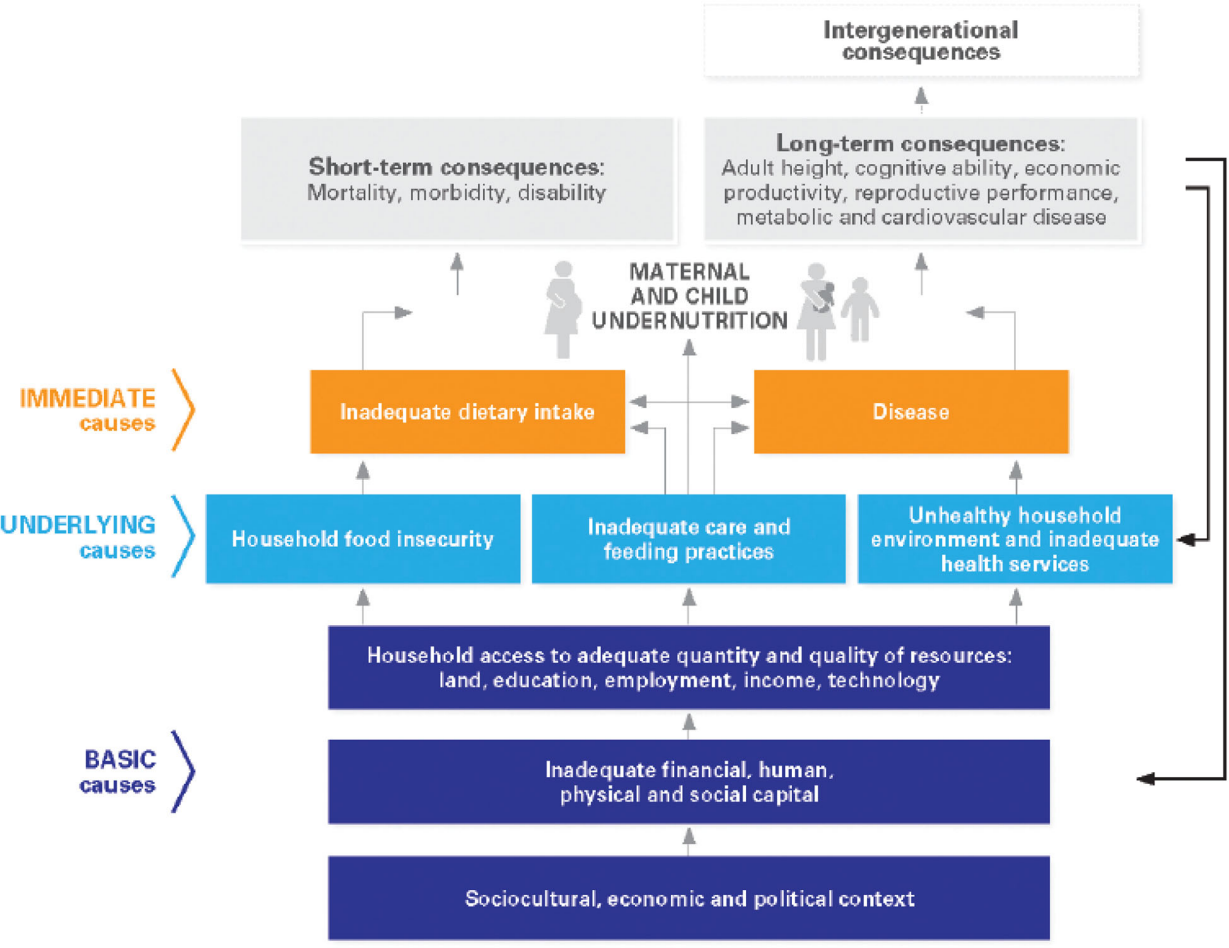
UNICEF conceptual framework of the determinants of child undernutrition

Studies on child undernutrition in individual countries or sub-regions within a country in South Asia have highlighted potential determinants that are consistent with the UNICEF conceptual framework, as part of UNICEF Nutrition Strategy for improving child nutrition (Fig. 1) [22]. The UNICEF conceptual framework on child undernutrition supports the development of interventions from a multi-factorial and multidimensional perspective, moving from micro to meso to macro levels. The framework includes the immediate, underlying, and basic determinants of child undernutrition and outlines that all these factors are interlinked and influence each other. The basic causes address the macro-systemic level challenges reflecting the structural and political processes which result in inadequate financial, human, physical, and social capital that influence household access to adequate quantity and quality of resources. The underlying causes focus on household food security, inadequate care and feeding practices, unhealthy household environment, and inadequate access to health services. The immediate causes of undernutrition such as inadequate food intake and disease are influenced by the basic and underlying causes [22]. The conceptual framework supports the development of multi-factorial interventions and has been modified for specific geographical contexts or with a focus on interventions [23, 24].

At present, there is no study that has collectively and systematically analysed the most consistent factors associated of child undernutrition, including of micronutrient deficiencies along with stunting, wasting, and underweight, across the entire South Asia region. There is also limited evidence that has analysed qualitative evidence (published qualitative articles and grey literature including programme reports and evaluation reports) which has resulted in a limited understanding of the factors of child undernutrition. This mixed-methods review will look at child undernutrition, including stunting, wasting, underweight and micronutrient deficiencies of iron, vitamin A, and zinc, assess its driving factors and identify priorities across the eight countries of South Asia.

#### Aim of this review and its public health significance

The review is necessary to appraise, synthesise, and summarise the literature on child undernutrition in South Asia. Most studies till date have focused either on undernutrition or on micronutrient deficiencies, and not both. This proposed review will look at various forms of undernutrition in children under 5 years of age, as defined in the scope of the review. It will create an evidence base that looks at multiple faces of macro and micro child undernutrition. It aims to contribute to the growing body of evidence needed to prioritise actions which are focused on the immediate, underlying, and basic determinants of child undernutrition. This review will include grey literature documents, including programme and evaluation reports and working papers. This research is also timely to generate evidence that will contribute to the policy discourse and to the Sustainable Development Goals (SDGs), especially Goal 2: end hunger, achieve food security and improved nutrition, and promote sustainable agriculture [25]. Findings from this study can be used to inform future programme and policy and also for knowledge enhancement and to drive region-specific interventions which could lead to a decline in child undernutrition within the region.

#### Review question

To assess the past drivers and identify priorities for child undernutrition including stunting, wasting, and underweight and micronutrient deficiencies of iron, vitamin A, and zinc, across eight South Asian countries.

Past drivers of undernutrition will focus on structural, underlying, and immediate determinants of child undernutrition to generate evidence for prioritising and planning for preventive action and identifying high-risk clusters of the population at the regional level.

## Methods

### Study design

This protocol is informed by the standard Preferred Reporting Items for Systematic Reviews and Meta-Analyses Protocols (PRISMA-P) reporting guidelines [26]. The PRISMA-P checklist is attached to this manuscript as Additional file 1. This mixed-methods review will look at both quantitative and qualitative evidence including peer-reviewed quantitative, qualitative, and mixed-methods studies and grey literature including programme reports and evaluation reports. The following criteria will be applied for inclusion.

### Eligibility criteria

#### Participants

Children under 5 years of age and those residing in a South Asian country including Afghanistan, Bangladesh, Bhutan, India, Maldives, Nepal, Pakistan, and Sri Lanka, as categorised in the United Nations geographical regions [27] will be included in the review. Any studies outside the defined population will be excluded from the review.

#### Intervention focus and design

Studies to be included in this systematic review will be those that focus on stunting, wasting and underweight and micronutrient deficiencies of iron, vitamin A, and zinc in a South Asian country. However, studies that look at child over nutrition, obesity, or overweight will be excluded.

Both published and unpublished literature [28, 29] will be included. Intervention designs of interest will be intervention studies (both randomised controlled trials (RCTs) and quasi-experimental studies), observational studies (e.g., longitudinal studies, case-control, and cross-sectional studies) as well as qualitative and mixed-methods studies. Unpublished grey literature will include programme and evaluation reports, and working papers. Grey literature such as editorials, dissertation and thesis, conference abstracts, opinion pieces, news articles, books, and book reviews will not be included. The grey literature sources included are based on the most frequently cited grey material in reviews [30]. Inclusion of programme and evaluation reports to the study will serve as a useful source to complement the findings from peer-reviewed literature. Due to resource restrictions, only studies published in English will be included. Studies published between January 2000 and September 2019 will be considered for review because this was the beginning of the Millennium Development Goals (MDGs) and will aid in tracking the progress of the region in line with the MDGs [31]. Aligning the base year of searching with the MDGs allows the outcomes to be more relevant within the international development policy framework. The MDGs created momentum across governments worldwide and formed an internationally agreed blueprint that most countries and leading multilateral and development institutions became signatories. The momentum created by MDGs needs to be sustained through the SGDs. Hence, the proposed systematic review is timely to provide a historical perspective of coordinated international efforts to end child undernutrition and to identify priorities for the SDGs.

#### Outcomes of interest

Outcomes will include factors and determinants of various forms of child undernutrition and other related outcomes including the following:

Primary outcome: Studies will be included in the review if they report about any one form of child undernutrition including stunting, wasting and underweight and the three micronutrient deficiencies with global significance in South Asia, namely, iron deficiency anaemia, vitamin A, and Zn [12, 13]. Stunting (height-for-age) is an indicator of linear growth retardation and cumulative growth deficits in children. Wasting (weight-for-height) measures body mass in relation to height and describes current nutritional status. However, underweight (weight-for-age) is a composite index of height-for-age and weight-for-height [32]. This systematic review focuses on children with a Z-score below minus two standard deviations (− 2 SD) from the median of the World Health Organisation (WHO) reference population [32, 33]. Anaemia measures in children 6–59 months will be of haemoglobin < 110 g/L, moderate anaemia of haemoglobin 70–99 g/L and severe anaemia of haemoglobin < 70 g/L [34]. Vitamin A deficiency, including both serum retinol concentrations < 10 μg/dL and subclinical vitamin A deficiency (serum vitamin A < 20 μg/dl) will be considered [35, 36]. Zinc deficiency will be considered as recommended by the International Zinc Nutrition Consultative Group [37, 38].

Secondary outcome: the secondary outcomes for this review will include low birth weight, intrauterine growth restriction, maternal body mass index, and maternal iron deficiency anaemia.

#### Search strategy

The search strategy of this protocol is designed to be as extensive as possible to identify all eligible studies, which will subsequently be refined according to the criteria outlined above. A multi-step search approach will be used to retrieve relevant studies from five academic databases. The databases that would be searched using a variety of subheadings and free-text terms:

1. CINAHL
2. EMBASE
3. PubMed
4. PsycINFO
5. Scopus

A combination of the above-mentioned subheadings and free-text words will also be used to search grey literature in key organisations websites, as listed below. This list might be reviewed when undertaking the research.

1. 3ie impact assessment
2. Action Against Hunger
3. Bills and Melinda Gates Foundation (BMGF)
4. Department for International Development (DFID)
5. International Food and Policy Research Institute [10]
6. Medecins Sans frontiers (Doctors without Borders)
7. Nutrition International
8. Save the Children
9. UNICEF
10. World Bank
11. World Food Program
12. World Health Organisation (WHO)

In addition, a lateral approach involving a review of reference lists in relevant papers/reviews will be undertaken. Forward-citation searches will also be undertaken. Search engines such as Google Scholar will be searched in conclusion to include any relevant articles and reports.

Search terms:

Child preschool [MeSH/Subheading] OR infan* [MeSH] OR under-five* OR preschool* OR paediatr* OR bab*

AND

Child malnutrition [MeSH/Subheading] OR Malnutr* [MeSH/Subheading] OR undernutr* [MeSH/Subheading] OR underweight [MeSH/Subheading] OR malnourish* OR undernourish* OR stunt* OR wast* OR “acute malnutrition” “OR chronic malnutrition” OR “micronutrient malnutrition” OR “micronutrient deficiency” OR “vitamin A deficiency” OR “iron deficiency” OR “iron deficiency aneamia” OR Anaemia OR “zinc deficiency”

AND

Caus* [MeSH/Subheading] OR Factor* OR determinant* OR correlate* OR “risk factor” OR multifactorial caus*OR priorit*

AND

“South Asia*” or “Southern Asia*” or Afghan* or Bangladesh* or Bhutan* or India* or Maldives or Nepal* or Pakistan* or Sri Lanka*These search terms provided are a combination of free-text words and the MeSH terms. The MeSH terms were tested in PubMed and were reviewed by all the authors. The School librarian was also consulted to finalise the search strategy. This search syntax has been tested and yielded a manageable number of records. The authors expect around 100–150 studies to be included in the final inclusion.

### Data collection

#### Study selection process

Studies yielded in the search will be imported into EndNote. Endnote software will be used to remove duplicates. The selection of studies will be done in three steps. Firstly, titles will be screened to remove any obviously irrelevant studies followed by the screening of abstracts to confirm eligibility and relevance. After this initial selection, full texts of studies will be reviewed for final inclusion. Final studies selected for full-text screening will be recorded and the reason for their exclusion will be documented in an MS excel sheet. This process will be undertaken independently by two researchers (NW and AR) and any disagreements will be resolved by the third researcher (KA).

#### Data extraction

The data extraction will comply with the Preferred Reporting Items for Systematic Reviews and Meta-Analyses guidelines [26]. Data extraction for studies for final included studies will be done using a piloted form including mainly: study details (such as author’s name, year of publication) study design, intervention type, study characteristics (including sample setting, population), and driver of child undernutrition and identified priorities, as documented in Table 1. This process will be undertaken independently by two researchers (NW and AR) and any disagreements will be resolved by the third researcher (KA).

**Table 1:** Data extraction form

Data to be extracted	
Authors	
Year of publication	
Study design	
Aims and objectives specified (Y/N)	
Ethics approval (Y/N)	
Intervention	
• Type	
• Duration	
Study methodology quant/qual/mix	
Population/participants (number, other characteristics)	
Method of data collection	
Outcomes	
• Stunting	
• Wasting	
• Underweight	
• Macronutrient deficiencies	
Factors of undernutrition	
• Structural determinants	
• Underlying determinants	
• Immediate determinants	
Quality appraisal	

#### Assessment of methodological quality

Final included studies will be assessed for methodological quality prior to inclusion in the review. The Critical Appraisal Skills Programme (CASP) will be used to assess the methodological quality of qualitative studies [39], randomised controlled trials [40], and observational studies such as longitudinal studies [41] and case-control studies [42]. CASP tool is a widely accepted and used tool to assess the quality of different study designs [43–45]. Mixed-methods studies will be assessed based on the MMAT (mixed-methods appraisal tool) by Pluye and colleagues. Grey literature will be appraised with the AACODS tool that looks at authority, accuracy, coverage, objectivity, date, and significance [46]. All studies will be appraised as having a high, medium or low quality and the overall quality of the body of evidence will be used using the Grading of Recommendations Assessment, Development and Evaluation (GRADE) approach [47]. Quality of all studies will be independently appraised by two researchers (NW and AR) and any disagreements will be resolved through discussions. Any further discrepancies will be independently reviewed by the third researcher (KA).

#### Data synthesis

Due to the heterogeneity and variation of the studies to be reviewed—especially the study methods, measurements, and outcomes—it might not be possible to determine the data synthesis methods priori. A narrative synthesis, a meta-ethnography, or a meta-analysis, as appropriate for the nature of the data retrieved, will be undertaken to understand the factors of and priorities for child undernutrition. The UNICEF conceptual framework of the determinants of child undernutrition will be used to frame the findings that enable to identify relationships between and across various determinants of child undernutrition and identify priorities.

#### Summary measures

Primary outcome measures will include the factors associated with stunting, wasting, underweight, iron deficiency anaemic, vitamin A deficiency, and zinc deficiency. Secondary outcome measures will include the factors associated with birth weight, intrauterine growth restriction, and maternal iron deficiency anaemia, as well as maternal nutrition status.

## Discussion

There is increasing awareness and concern about child undernutrition especially the extent of stunting, wasting, underweight, and micronutrient deficiencies amongst children under 5 years of age, particularly in South Asia. This is also of mounting interest for governments and policy-makers, given its long-term consequences. Though there is ample literature on general nutritional status, there is limited synthesised evidence that collectively looks at micronutrient deficiencies along with stunting, wasting, and underweight. This systematic review will add to the extant literature by synthesising the evidence on the multiple faces of child undernutrition, including stunting, wasting, and micro deficiencies of iron, vitamin A, and zinc across the eight South Asian countries. Findings of this review can inform policies and strategies to combat child undernutrition in the region.

One of the major strengths of this review is the use of a systematic and transparent approach, employing UNICEF conceptual framework for data synthesis. We anticipate some limitations in the review, such as the exclusion of studies published in other languages than English. Exclusion of such studies could lead to missing key literature generated by non-English-speaking researchers and regional organisations. This limitation is addressed, partly, by expanding the search to include relevant grey literature documents from international organisations and by searching google scholar. This review will not look at child over nutrition, obesity, or overweight. This review will only include studies that were published before the year 2000 and could miss relevant articles published before this time frame that might have contributed to this review. In the final review, any discrepancies between the review and the protocol will be explained. We will ensure that the final manuscript is an accurate and transparent account of the review, and that no important aspects of the review will be omitted.

### Target audience and dissemination plans

This work has been conducted as part of a PhD dissertation at the School of Social Sciences and Psychology, Western Sydney University. The findings of the review will be shared at conferences and other public forums. Target audience for this review are researchers, practitioners, and policy-makers.

### Study registration

This systematic review has been registered with PROSPERO—the International Prospective Register of Systematic Reviews, registration number: CRD42018112696.

## Additional file

Additional file 1: PRISMA-P checklist. (DOCX 33 kb)

## Data Availability

Not applicable.

## Abbreviations

AACODS: Authority Accuracy Coverage Objectivity Date and Significance
BMGF: Bills and Melinda Gates Foundation
CINAHL: Cumulative Index to Nursing and Allied Health Literature
DALYs: Disability Adjusted Life Years
DFID: Department for International Development (United Kingdom Government)
EMBASE: Excerpta Medica DataBASE
IFPRI: International Food and Policy Research Institute
LMICs: Low- and middle-income countries
MDGs: Millennium Development Goals
MEDLINE: Medical Literature and Retrieval System Online
MMAT: Mixed-Methods Appraisal Tool
PRISMA-P: Preferred Reporting Items for Systematic Reviews and Meta-Analyses Protocols
PROSPERO: International Prospective Register of Systematic Reviews
RCTs: Randomised controlled trials
SDs: Standard deviations
UNICEF: United Nations International Children’s Emergency Fund
VAD: Vitamin A deficiency
WHO: World Health Organisation
